# Anticipated Barriers and Facilitators to Engaging in a Novel Virtual Reality Program for Lower Phantom Limb Pain

**DOI:** 10.1089/jmedxr.2024.0047

**Published:** 2025-03-31

**Authors:** M. Crooks, M. Smith, K. Reynolds, E. Hammond, P. Gross, L. Pankratz, R. El-Gabalawy

**Affiliations:** ^1^ Department of Psychology, University of Manitoba, Winnipeg, Canada.; ^2^ National Research Council of Canada, Winnipeg, Canada.; ^3^ Department of Physical Therapy, Max Rady College of Medicine, Rady Faculty of Health Sciences, University of Manitoba, Winnipeg, Canada.; ^4^ Shared Health Manitoba, Canada.; ^5^ Department of Clinical Health Psychology, Max Rady College of Medicine, Rady Faculty of Health Sciences, University of Manitoba, Winnipeg, Canada.; ^6^ Department of Anesthesiology, Perioperative and Pain Medicine, University of Manitoba, Winnipeg, Canada.; ^7^ Department of Psychiatry, University of Manitoba, Winnipeg, Canada.; ^8^ CancerCare Manitoba, Winnipeg, Canada.

**Keywords:** phantom limb pain, lower limb amputation, virtual reality, graded motor imagery, barriers, postoperative

## Abstract

While there is promising evidence to suggest certain virtual reality (VR) programs and graded motor imagery (GMI) can independently be administered to treat phantom limb pain (PLP) in people with lower limb amputations (LLAs), there are many barriers preventing their implementation. Long outpatient wait times prevent treatment access in the early postoperative period following amputation, when PLP is the most severe. The integration of GMI in VR offers the opportunity to improve PLP treatment access in the acute period by facilitating self-administration. Accordingly, the present multidisciplinary team used a multi-methods approach to assess a novel head-mounted VR GMI prototype and evaluate areas of improvement according to patient feedback. Twelve people with unilateral LLAs recruited from outpatient physiotherapist and prosthetic clinics were asked to trial the program in a single intensive session. Afterwards, participants completed a semi-structured interview to reflect on barriers and facilitators they expect would affect their use of the VR GMI program. They also completed psychometrically validated self-report questionnaires (including the User Engagement Scale and Presence Questionnaire) that inquired about the engagement and immersion facilitated by the program. Reflexive thematic analysis suggests VR and treatment expectations, in addition to individual priorities, motivation, and resources, may affect one’s willingness to use the VR GMI program in the acute period following amputation. Meanwhile, descriptive analysis demonstrates that while the VR GMI program is considered immersive, more focused attention needs to be facilitated to increase engagement. In line with these findings, future development will prioritize embedding psychoeducation to prime realistic expectations about the intervention. These developments will improve the chances of implementing the VR GMI program in clinical settings following a larger study to assess its efficacy in the hospital following amputation. Results may be transferred to our broader understanding of how VR interventions may be implemented in acute postoperative settings.

## Introduction

Approximately 70% of people with lower limb amputations (LLAs) experience phantom limb pain (PLP), where stabbing, burning, or throbbing pain is felt in the missing limb despite its absence.^1^ There are two promising nonpharmacological interventions for PLP: graded motor imagery (GMI) and virtual reality (VR) programs. GMI is an evidence-based approach involving mental visualization of the phantom limb through three tasks: left/right discrimination, explicit motor imagery, and mirror therapy (which is typically delivered by specialized health care professionals in outpatient settings).^2,3^ VR programs have also shown potential for PLP relief by providing virtual visual feedback similar to mirror therapy, which uses a mirror to create an illusion of the missing limb by reflecting the existing one.^4^ Additionally, VR offers gamification opportunities that can enhance patient engagement in phantom limb movement exercises.^5^ Both GMI and VR can increase patients’ sense of agency over their phantom limb, a critical factor in reshaping the brain’s response to amputation and reversing the maladaptive rewiring of the sensorimotor cortex implicated in PLP.^3,6–8^ VR’s immersive qualities may further enhance treatment efficacy and patient motivation.^9,10^ A recent meta-analysis suggests that implementing nonpharmacological interventions such as VR and/or GMI could yield more effective PLP relief than medication alone.^11^

Despite the potential of VR and GMI, studies exploring their clinical feasibility are limited. Early research highlights several barriers to their use in outpatient settings, including patients’ doubts about treatment efficacy, lack of in-home equipment, time demands, limited technical support, financial constraints, and clinician dependence.^12–14^ Accessibility challenges, such as travel difficulties and navigating outpatient care, further hinder PLP treatment access for people with LLAs.^15–17^ In Canada, the average wait time for outpatient pain specialists is over 6 months.^18,19^ Given that PLP is the most severe and responsive to intervention within 1 year following LLA, these treatment delays and barriers may have adverse consequences for PLP recovery.^1,20^ For PLP treatment to be accessible and utilized effectively, barriers to its implementation need to be recognized and mitigated.

To provide accessible, timely PLP care, our research team developed an innovative VR program that incorporates GMI principles, enabling individuals with LLAs to self-administer PLP treatment.^21^ This study aimed to refine our VR GMI prototype based on feedback from persons with lived experience and represents the first empirical investigation of a novel VR program embedding GMI. Our primary objective used qualitative methods to explore potential barriers and facilitators for using the VR GMI program at home and in the hospital (i.e., immediately postamputation, when the VR GMI intervention is intended for initial implementation). Our secondary objective used quantitative methods to assess participant engagement and immersion in the VR GMI program.

## Methodology

The present study employed a multi-method design. Recruitment was conducted between March 2023 and September 2023. People with LLAs living in the community trialed the VR program in an approximately 1.5-hour session at the National Research Council (NRC) in Winnipeg, Canada. Participants then completed psychometrically validated questionnaires, followed by a semi-structured interview, to describe their experience.

### Researcher description

The current research utilized reflexive thematic analysis, a qualitative approach that emphasizes the active role the analyst plays in theme generation.^22,23^ By completing a researcher description, the principal researcher practices reflexivity by acknowledging how her personal identities and upbringing will affect how she perceives participants (and vice versa) to promote transparent and rigorous qualitative research.

The principal researcher is a young, white, cisgender woman who has spent her life in institutional education. She acknowledges the social privilege that accompanies her physical appearance and position as a master’s student in clinical psychology. Moreover, the principal researcher is nondisabled. Her distanced positionality may affect the depth to which participants feel they may disclose their experiences. The principal researcher also acknowledges her perspective will affect her understanding of her participants during interviews, and in turn, her interpretation of the findings. The principal researcher aims to be aware of these biases while understanding participant experiences to produce meaningful qualitative results within the context of her positionality.

### Participants

The research team aimed to recruit 10–15 participants via purposive sampling. This sample size estimate was generated following the analysis of the first two interview transcripts to ensure there was sufficient information power (i.e., density, quality, and breadth of the qualitative data^24^) to address the specific research aim, as recommended by prior research.^25^ Recruitment posters were distributed to relevant community organizations and medical clinics as well as social media. Willing health professionals also directly discussed the study with their patients and/or disseminated posters. People who were interested in participating phoned or emailed the research personnel directly or consented to provide their contact information.

The principal researcher contacted interested individuals via phone for screening. Potential participants were assessed for the following inclusion criteria: must have a unilateral leg amputation above, below, or through the knee or ankle; must be at least 18 years of age or older; must not be experiencing any hearing or vision loss that would affect interaction with a VR headset; must have experienced PLP or sensation either in the past or present; and must agree to be audio recorded. A $25 gift card was provided to participants as an honorarium.

Ethics approval (HE2022-0374) was obtained from the Research Ethics Board at the University of Manitoba.

### Procedure and measures

At their scheduled appointment, participants completed the sociodemographic and background questionnaire and responded to a semi-structured interview regarding their amputation and pain history. Brief verbal education around GMI and the operation of the VR headset, an Oculus Quest 2, was provided. Afterwards, participants proceeded to trial the VR intervention.

**FIG. 1.**
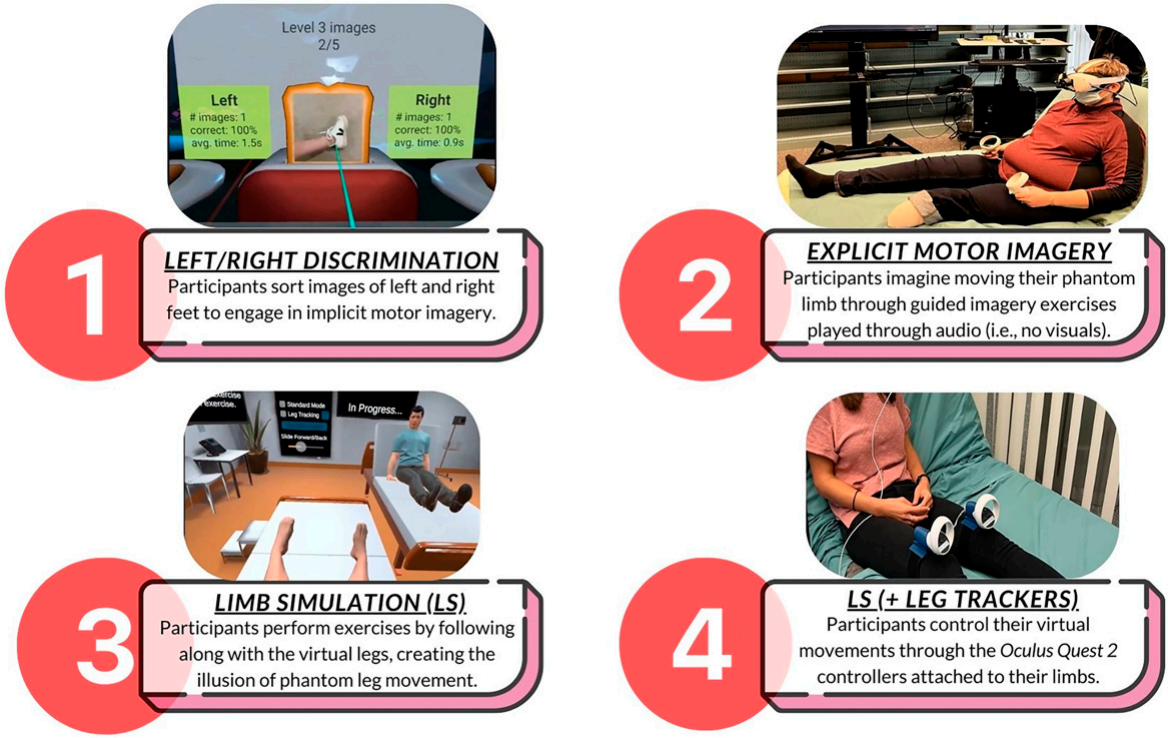
Snapshots of what participants see in the virtual reality program, developed according to graded motor imagery.

Within the VR, participants explored each of the three stages, including left/right discrimination, explicit motor imagery, and limb simulation (which can be administered with or without leg trackers), described further elsewhere.^21^ See Figure 1 for a visual overview of the intervention content or the Supplementary Appendix for a written description. The participants were explicitly instructed to vocalize any thoughts they had about the program while using it, which were recorded.

Following VR, participants completed the validated User Engagement Scale (UES^26,27^) and Presence Questionnaire (PQ^28^). The UES and PQ measured how engaging and immersive the participants perceived their overall experience in the VR program to be, respectively. The UES subscales include focused attention (defined as the degree to which one is cognitively invested in the VR GMI program; e.g., *“I lost myself in this VR experience”*), perceived usability (e.g., *“I could not do some of the things that need to be done in the VR”*), aesthetic appeal (e.g., *“I liked the graphics and images used in the VR”*), and reward (defined as the extent to which a participant finds the VR GMI program to be a worthwhile experience; e.g., *“I could continue this VR out of curiosity”*). The PQ subscales include involvement (defined as how involved the participant felt in controlling the VR GMI program; e.g., *“how much were you able to control events?”*), natural (defined as how natural or realistic movements in the VR GMI program felt; e.g., *“how much did your experiences in the virtual environment seem consistent with your real-world experiences?”*), and interface quality (defined as the degree to which one could interact with the VR GMI program without feeling distracted by the controls or visual display; e.g., *“how much did the control devices interfere with the performance of assigned tasks or with other activities?”*). Since UES scores are rated on a scale of 1–5 (with 3–4 referring to *no opinion* or neutral), any scores valued at less than 3 indicate poor engagement, while 5 indicates the highest rating of engagement. A similar interpretation applies to the PQ scores, rated on a Likert scale of 1–7 (with 4–5 referring to *no opinion* on presence).

Participants concluded the study with a semi-structured exit interview that ranged from approximately 10–20 min in length and was audio recorded. Each participant was asked whether they think they would be interested and able to use the VR GMI program in their home and in the hospital following their LLA.

### Data analysis

#### Qualitative analysis

All interviews were transcribed via Trint (an automated speech recognition transcription software) and exported as a Word document for analysis. For the reflexive thematic analysis, the principal researcher identified codes via comments in each of the Word documents and generated initial themes as bullet lists through an inductive approach. Initial themes were reviewed and recoded in collaboration with qualitative researchers (L.P. and K.R.) multiple times to correct postpositivist inclinations and increase qualitative rigor. Final themes and data extracts were reviewed and edited by the entire research team.

#### Quantitative analysis

Quantitative measures were descriptively analyzed using R software. Listwise deletion was used for any missing items. The data were then analyzed through descriptive statistics (including mean, standard deviation, and median) and visualization, such as boxplots (to identify outliers) and bar graphs (to communicate the frequency of favorable compared with unfavorable ratings of engagement and immersion).

## Results

### Sample characteristics

A total of 15 individuals were screened, and 12 (*n* = 12) participants were recruited. Of the recruited individuals, six (50%) reached out following a mass email from their prosthetic clinic, five (41.67%) were referred by their physiotherapist, and one (8.33%) was referred by the surgeon who performed their LLA. Two individuals did not proceed to informed consent following screening (not mobile enough to visit the NRC; withdrew due to preexisting anxiety). Another individual was excluded for having a bilateral amputation.

On average, participants were 51.25 years old (SD = 17.42), with ages ranging from 18 to 81. Most participants were male (66.67%), married (50.00%), had a college education (66.67%), and sustained a below-knee amputation (75.00%) 2–5 years ago (41.67%) due to atraumatic indications (such as cancer, ischemia, or infection; 91.67%). Eight (66.67%) participants reported current PLP of varying quality and intensity, describing the episodic pain as “electric,” “growing pains,” “numbness,” “sharp,” “shooting,” and tightness/binding of the foot. Nine (75%) participants reported current phantom limb sensations (i.e., self-reported nonpainful phantom phenomena), describing it as pins and needles, an urge to move, nerve jumpiness, awareness of the toes or calf, or “stepping on a Lego”. Five (41.67%) participants stated their PLP or phantom limb sensation interfered with their daily life. See Table 1 for an overview of the sample characteristics.

**Table 1.** Sample Characteristics (*n* = 12)

Age (years)	
18–29	2 (16.67%)
30–39	0 (0%)
40–49	4 (33.33%)
50–59	2 (16.67%)
60–69	2 (16.67%)
70–79	1 (8.33%)
80+	1 (8.33%)
Gender	
Male	8 (66.67%)
Female	4 (33.33%)
Marital status	
Married	6 (50.00%)
Single	3 (25.00%)
Divorced/separated	3 (25.00%)
Education level	
High school/GED equivalent	4 (33.33%)
College or higher	8 (66.67%)
Amputation type	
Above knee	3 (25.00%)
Below knee	9 (75.00%)
Time since amputation	
3 months–2 years	4 (33.33%)
2–5 years	5 (41.67%)
5 years+	3 (25.00%)
Reason for amputation	
Atraumatic (cancer, infection, ischemia)	11 (91.67%)
Traumatic (car accident)	1 (8.33%)
Current PLP	
Yes	8 (66.67%)
No	4 (33.33%)
Current phantom limb sensations	
Yes	9 (75.00%)
No	3 (25.00%)
PLP/sensation interference	
Yes	5 (41.67%)
No	7 (58.33%)
Previous VR use	
Yes	3 (25.00%)
No	9 (75.00%)

PLP, phantom limb pain; VR, virtual reality.

### Objective 1: Anticipated barriers and facilitators to VR GMI program use

**FIG. 2.**
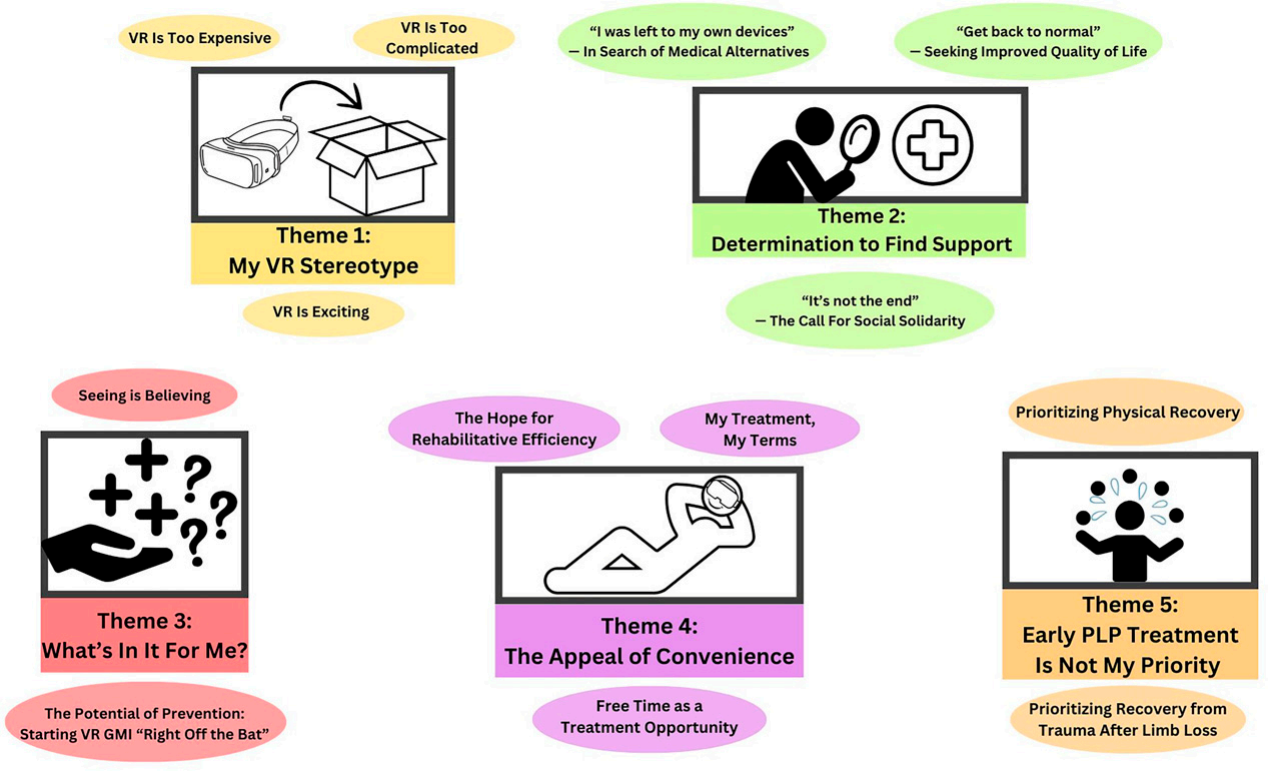
A visual overview of the 5 themes and the 14 subthemes as they pertain to potential barriers and facilitators to using the VR GMI program in the postoperative acute period following amputation. GMI, graded motor imagery; PLP, phantom limb pain; VR, virtual reality.

Average interview length was 15 min and 53 s (range = 9:51 to 20:11 min). The reflexive thematic analysis resulted in five themes and 14 subthemes. A detailed description of the themes and representative quotes can be found in Table 2. See Figure 2 for a visual overview of the themes and subthemes.

**Table 2.** Objective 1 Themes and Subthemes

Theme	Definitions	Supplemental quotes
Theme 1: My VR stereotype	Defined as how a participant’s preconceived notions or expectations of VR technology affect their willingness to use the VR GMI program as a treatment.	
Subtheme 1: VR is too complicated.	Defined as the assumption that the VR GMI program is too complicated to independently understand and use.	*When it comes to gadgets and electronics and all that stuff, I often I just kind of block it out, right. And say, “Oh, I can’t do this right”. [P2]* *I guess I don’t have the foresight for seeing what you would use the VR for... This is how new I am to this stuff. Because I, I do understand the questions that I ask are probably pretty simplified to you guys, but to me… [P3]* *Especially (useful) if someone was younger and they already knew the technology… that would actually be a fun way to rehab [P8]*
Subtheme 2: VR is exciting.	Defined as the expectation for VR technology to be exciting through realism and immersion. Satisfaction with the VR GMI program varies depending on whether this expectation is met.	*When I go play VR games, like I get lost in the game and I don’t actually feel my phantom pain anymore because I’m moving around in the game. [P4]* *You’re still, like, at your home, but, like, it feels like you’re somewhere else. And you can do more things [in the VR] [P12]*
Theme 2: Determination to find support	Defined as the motivation to pursue supplementary outpatient care (such as the VR GMI program) to assist with rehabilitation following amputation.	
Subtheme 1: “I was left to my own devices”— In Search of Medical Alternatives.	Defined as the motivation to search for treatment alternatives following amputation due to dissatisfaction with traditional medical care.	*I’d rather use [this treatment] than take medication because I don’t like medication. Like I’m not on anything anymore and I’ve worked hard to get to that stage. [P1]* *Like everything, for some people, it works, for other people, it doesn’t, right? So that’s always the risk you take with anything. That’s why I went with the revision [surgery], because I was told it would be life altering. I’m not giving up yet, but it certainly hasn’t quite done what I anticipated it would do. [P2]* *It was sort of like, just suck it up and deal with it… Like I tried mirror therapy at home, it doesn’t work. [P4]*
Subtheme 2: “Get back to normal”—Seeking improved quality of life.	Defined as the motivation to pursue alternative medical care so one may return to the quality of life they had before their amputation.	*Another option for treatment… I’m fairly driven to get my life back to as normal as I can [P7]* *I would have wanted to because I did want to restart my life [P8]* *I’m always outdoors. . . . That’s what helps me to try to do things more, to try to be more independent. I feel like I was a burden to my family. [P11]*
Subtheme 3: “It’s not the end”—The call for social solidarity.	Defined as the motivation to seek validation and understanding through social support following amputation.	*I actually do think there should be more programs and maybe there are. I just don’t know about them. But more programs available. Supports, just like cancer support and stuff, but amputation support where you could have like get to know other people and, and have that support system. [2]* *Just giving people access… [I learned] creative visualization with the cancer groups… but I don’t think there was anything for amputees back then. I don’t know how long they’ve been doing stuff [research] like this. [10]* *Games [would be helpful] … have just a [virtual] person [who also does not have a limb] you can tell things to… it’s hard to tell a stranger [therapist] how you feel I guess, but this is just a VR thing. You can just tell it. [12]*
Theme 3: What’s in it for me?	Describes the necessity of understanding how and why VR GMI treatment is beneficial during or before administration to motivate adherence.	
Subtheme 1: Seeing is believing.	Defined as the motivation a participant gains from achieving experiential benefit from or progress in the VR GMI program. For example, the participant may experience PLP reduction, increased physical strength, or conquer challenges in the VR GMI program.	*If there’s a high score board or if there’s like something that you can do daily to maybe even beat or better yourself, then you can set goals… that adds replayability, making it more of a tradition or long standing daily sort of thing that you can do. [P5]* *For me, I have limited phantom pain or sensation… I would [use the VR] for awhile, I think. And I would probably just forget and stop because it’s summer and I’m busy [P6]* *Expanding the idea of retraining the brain… to fit into stuff I’m actually doing. For most of my life, I’ve been very active. Go do activities [in the VR] with motions that are also involved in a golf swing… that would be useful, I think [P10]*
Subtheme 2: The potential of prevention—Starting VR GMI “right off the bat”.	Defined as the interest in using the VR GMI program immediately following amputation to improve PLP treatment effectiveness.	*I think if you got [this VR program] in the hospital right off the bat, I think it would probably help a lot… I was sort of just left to my own devices as to what to do, but to have this post-op would have been something that probably would have helped me a lot better than what I didn’t have. [P4]* *I wish there had been something like this when I first lost my leg [P8]* *If you started with [treatment] right off the bat it might be easier… I just shut down and went to bed… there’s nothing I could do [about the pain] [10]*
Theme 4: The appeal of convenience	Describes how the effort required to use the VR GMI program influences a participant’s willingness to routinely use it.	
Subtheme 1: The hope for rehabilitative efficiency.	Defined as the motivation to combine PLP treatment through the VR GMI program with physiotherapy and other physical exercises to be more efficient with the time spent recovering following amputation.	*If I had (the VR program) in my hospital bed, I could have done more of (rehab) and it would have been more beneficial [P8]* *It would be cool to be able to do more. You know, I can get--like right now I’ve lost like a lot of my physical fitness and this kind of thing that also helps with [PLP] and makes you stronger and get going. [P9]* *I think it would be a step before physiotherapy. Like in terms of building up your energy because it’s physically demanding once you first start with the physio… if you’re not doing nothing. [P11]*
Subtheme 2: My treatment, my terms.	Defined as the ease with which one could use VR GMI at the time and location of their choosing.	*[the VR] could go with you wherever you want to go, you can take it with you [P2]* *if you can use it before bed or, you know, in the morning or whatever. Yeah. if it’s just a matter of putting the headset on and the controllers. Mm hmm. Yeah, that would be totally usable. [P10]*
Subtheme 3: Free time as a treatment opportunity.	Defined as the interest in self-administering VR GMI treatment during free time, such as in the hospital following amputation.	*Sitting in a hospital bed for days on end can be really mentally taxing and draining… you’re kind of just finding ways to distract yourself while you’re in the healing process. This would give you something to do. [P5]* *Sitting in the hospital for any period of time is extremely boring and mentally taxing… a change of pace would be beneficial. [P7]* *I could see myself being bored, you know, in the afternoon and just hooking it up [P8]*
Theme 5: Early PLP treatment is not my priority	Describes the reservations participants have about self-administering nonpharmacological PLP treatment so soon after their amputation, given other priorities for recovery.	
Subtheme 1: Prioritizing physical recovery.	Defined as how a participant’s physical healing might affect their willingness to use the VR GMI program, particularly if they were offered the treatment in the hospital immediately following their amputation.	*Supposed to go through 16 weeks of training and I finished and I got discharged in 4… good to go do some stairs. But I saw people that were there… six weeks after their surgery. So everyone is different. [P1]* *There’s a healing process that you have to go through. You have to make sure that the skin is strong enough in order to put, like, weight on that area as well. [P5]* *I think you could use it after surgery if you just have that, you know, the bone [isn’t in the way], just amputation. [P12]*
Subtheme 2: Prioritizing recovery from trauma after limb loss.	Describes how the psychological trauma of limb loss could make focusing on PLP harmful or undesirable in the acute period following amputation.	*I think it might be difficult to use right out of having the surgery because when you have the surgery, the first thing you want to do is try and put weight on the limb that is not there… you can kind of fall and hurt yourself. And if you’re building that association with there potentially being something there… you can cause a bit more trouble that way. [P5]* *[the leg trackers] should be done under supervision… I do almost feel like I have my leg back… Yeah it could be like how people go to drugs and stuff like that. [P12]*

GMI, graded motor imagery.

#### Theme 1: My VR stereotype

The *My VR stereotype* theme encompasses the implicit expectations participants have about VR technology and whether these preconceived notions complement one’s assumptions about themselves. Participants have developed stereotypes of what using VR should entail and whether they are capable of it. Each of the three subthemes describes a different VR stereotype. First, participants assumed VR technology would be too expensive for them to use for PLP treatment, particularly considering the other costs associated with amputation (e.g., prosthetics). Second, participants assumed the VR GMI program would be too complicated for them to use on their own, often referring to older age as the reason for their reliance on support for set-up and operation.

The last VR stereotype participants assumed was that VR should be exciting. Given its novelty, participants had high expectations as to what VR should be able to accomplish through immersion and realism. For participants whose VR expectations were met, they described feeling transported by the simulation. For example, participant #11 (P11) said:

*(The VR program) kind of reminds me of the movie Avatar when he hooks up to the machine… (even though) you know deep down you don’t have a foot.*

Meanwhile, other participants were let down by their VR GMI experience. For example, P7 had previous experience with VR, and they were disappointed by the VR GMI program in comparison:

*Like some of the VR sets I’ve played with in the past. Like, wow, that is trippy. This was– I hate to use the word, but amateurish. It didn’t have the same production to it.*

These quotes illustrate how different expectations can have a significant effect on one’s willingness to use the VR GMI program.

#### Theme 2: Determination to find support

*Determination to find support* embodies the resolve to pursue any treatment that may assist with rehabilitation following amputation. Such resolve is a testament to the resilience participants must exercise to receive adequate care. Three subthemes describe the motivation behind participants’ pursuit of support. First, participants expressed disappointment with the current PLP medical supports, stating they were prompted to seek alternatives after they were “left to their own devices” [P4]. Second, participants were motivated to use the VR GMI program to reattain the quality of life they enjoyed prior to their amputation (i.e., “get back to normal”; P1, P7). Lastly, participants described being determined to find social solidarity to improve their psychosocial health. Many participants sought connections with peer groups, while others communicated a desire to connect meaningfully with their medical team (even through VR). Participants indicated that these social connections helped them feel more hopeful, as though their amputation “is not the end” [P10]. This theme suggests people with LLAs would maintain their use of novel treatments such as the VR GMI program for a variety of different reasons, including ones that were not intended for the VR GMI program (e.g., social solidarity).

#### Theme 3: What’s in it for me?

This theme acknowledges that motivation to use the VR GMI program will primarily depend on whether one benefits (or at the very least, anticipates a benefit) from its use. Participants implied seeing is believing; in other words, they suggested experiencing progress in the VR GMI program would motivate their treatment adherence. While progress often meant experiencing reductions in their PLP, some also suggested they would be motivated by conquering gamified challenges in the program or increasing their physical strength. Participants also endorsed being motivated by the potential of prevention by starting VR GMI “right off the bat” [P4, P10]. Some participants demonstrated an understanding of the neuroscience of PLP and why early intervention may reduce their suffering in the long term, in line with GMI psychoeducation briefly administered prior to their trial of the VR GMI program (e.g., by “retraining the brain early”; P6). *What’s in it for me* highlights the necessity of people with LLAs understanding the reasons to engage in the VR GMI program to motivate treatment adherence.

#### Theme 4: The appeal of convenience

*The appeal of convenience* describes how a participant’s willingness to use the VR GMI program is motivated by how easy the treatment is to use and integrate into their daily life. It includes three subthemes that elaborate on what convenient VR GMI administration should entail. For example, participants expressed the desire to combine PLP treatment with physiotherapy to increase their rehabilitative “efficiency” [P10] and save time on individual exercises. Furthermore, participants were motivated to use the VR GMI program in their home because they could self-administer it according to their own preferences, whether it be “lying in bed or sitting on the couch” [P5]. Lastly, participants were enthusiastic about using the VR GMI program when they were “bored” [P6, P7, P8], such as during their hospital stay. The capacity of the VR GMI program to be used independently gives participants the freedom to administer the treatment at their own convenience.

#### Theme 5: Early PLP treatment is not my priority

While many participants voiced positive impressions of early VR GMI intervention to prevent PLP or boredom while in recovery, others voiced concerns about potential dangers to its acute use. *Early PLP treatment is not my priority* encapsulates the belief that other aspects of physical and psychological recovery need to take precedence over PLP. For example, when participants considered early in-hospital use, they were concerned about their residual limb (i.e., physical limb at the amputation site) and whether it could handle the treatment tasks. Some participants also believed immediate PLP treatment could be traumatic and negatively affect their ability to cope with the loss of their limb. This concern manifested in different ways, with some participants worried about how creating the illusion they still had their leg would affect their psychological adjustment following their amputation. For example, P10 stated:

*You have to be sensitive at the hospital… it’s trauma. Most people are trying to figure out how they’re going to pay their bills in hospital, never mind whether they can still feel their foot.*

These concerns highlight the potential dangers of introducing the VR GMI program before patients have processed the trauma of LLA and that PLP may not be an appropriate priority for every patient immediately following amputation.

#### Objective 2: Engagement and immersion

All but one participant completed the UES and PQ (excluded due to lack of time and difficulties with writing), resulting in *n* = 11 respondents. Only one item was missing and removed from the mean score calculation for the aesthetic appeal subscale.

**FIG. 3.**
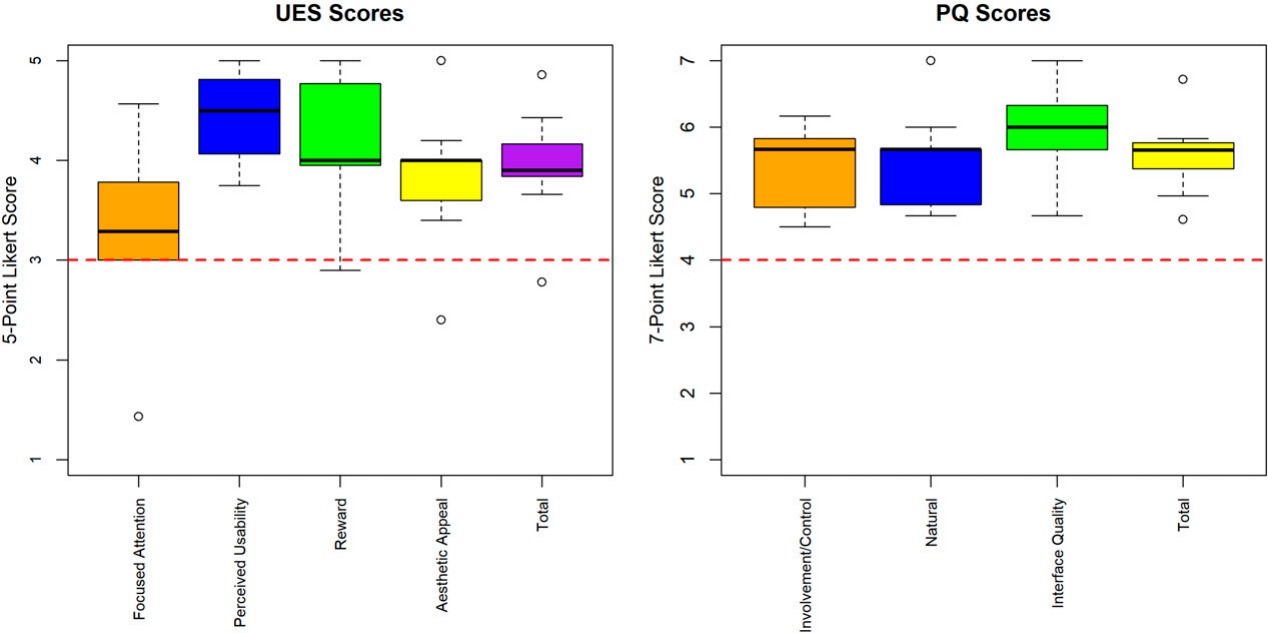
Displaying the boxplots for the User Engagement Scale (UES) and Presence Questionnaire (PQ) scores. The red dotted lines indicate the cutoff scores for an unfavourable rating of presence or engagement, respectively. Outliers are indicated by empty dots.

On average, participants provided neutral ratings for the focused attention (*M = 3.35, SD = 0.85*) and aesthetic appeal (M = 3.80, SD = 0.63) subscales, in addition to the total UES score (M = 3.96, SD = 0.51). Participants indicated favorable ratings for the rest of the engagement and immersion scores, including reward (M = 4.23, SD = 0.64), perceived usability (M = 4.44, SD = 0.45), involvement/control (M = 5.35, SD = 0.62), natural (M = 5.46, SD = 0.70), interface quality (M = 5.91, SD = 0.67), and total PQ (M = 5.57, SD = 0.53). Median UES and PQ scores were also calculated to account for outliers (see Fig. 3 for boxplots of the summary and subscale scores). In line with the mean ratings, these scores indicate participants agree the program is usable, rewarding, and immersive, but are more neutral about its aesthetic appeal and capacity to focus their attention.

**FIG. 4.**
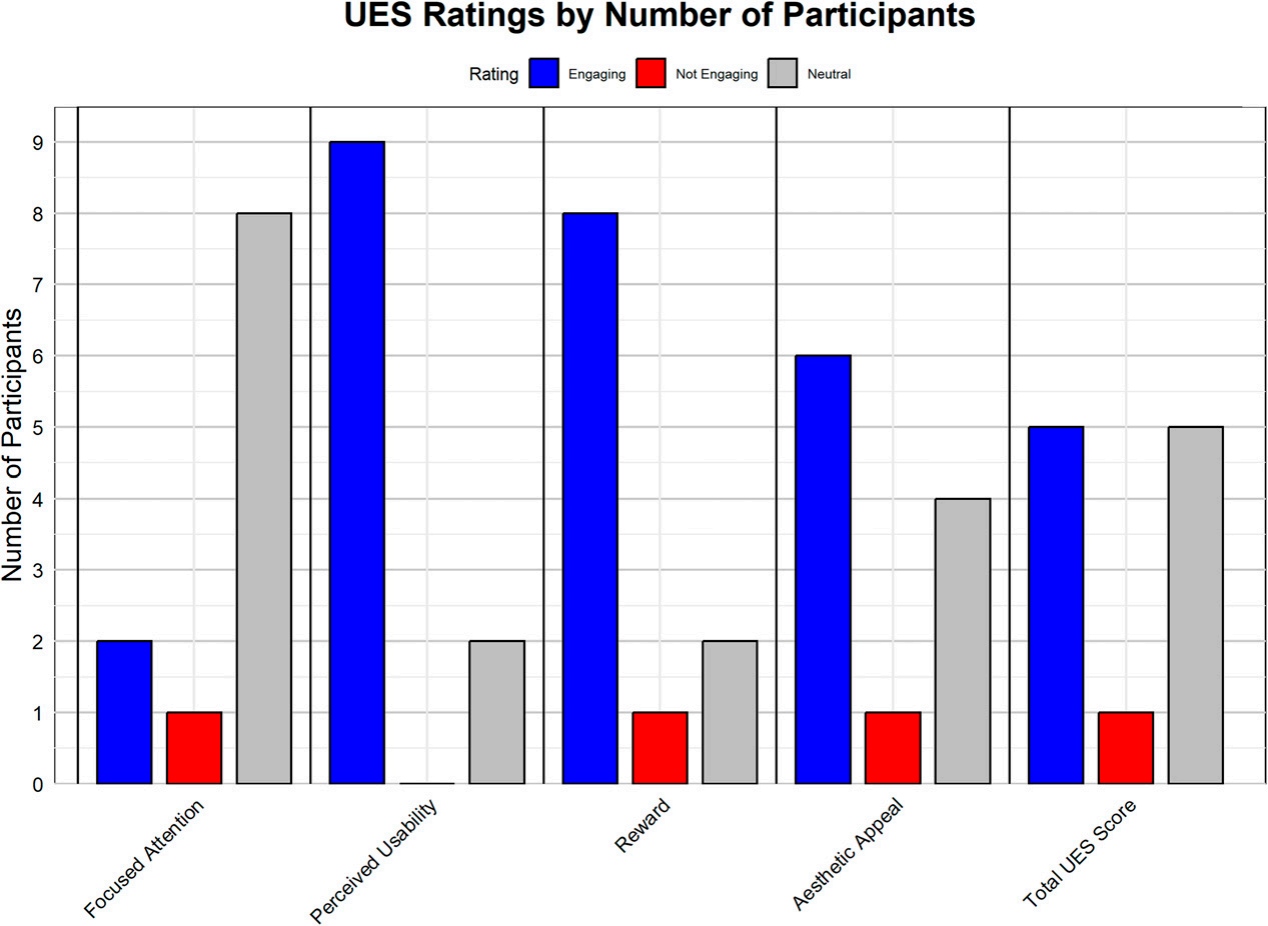
Describing the number of participants who gave an “engaging,” “not engaging,” or “neutral” rating of the intervention for each of the subscales on the User Engagement Scale (UES).

**FIG. 5.**
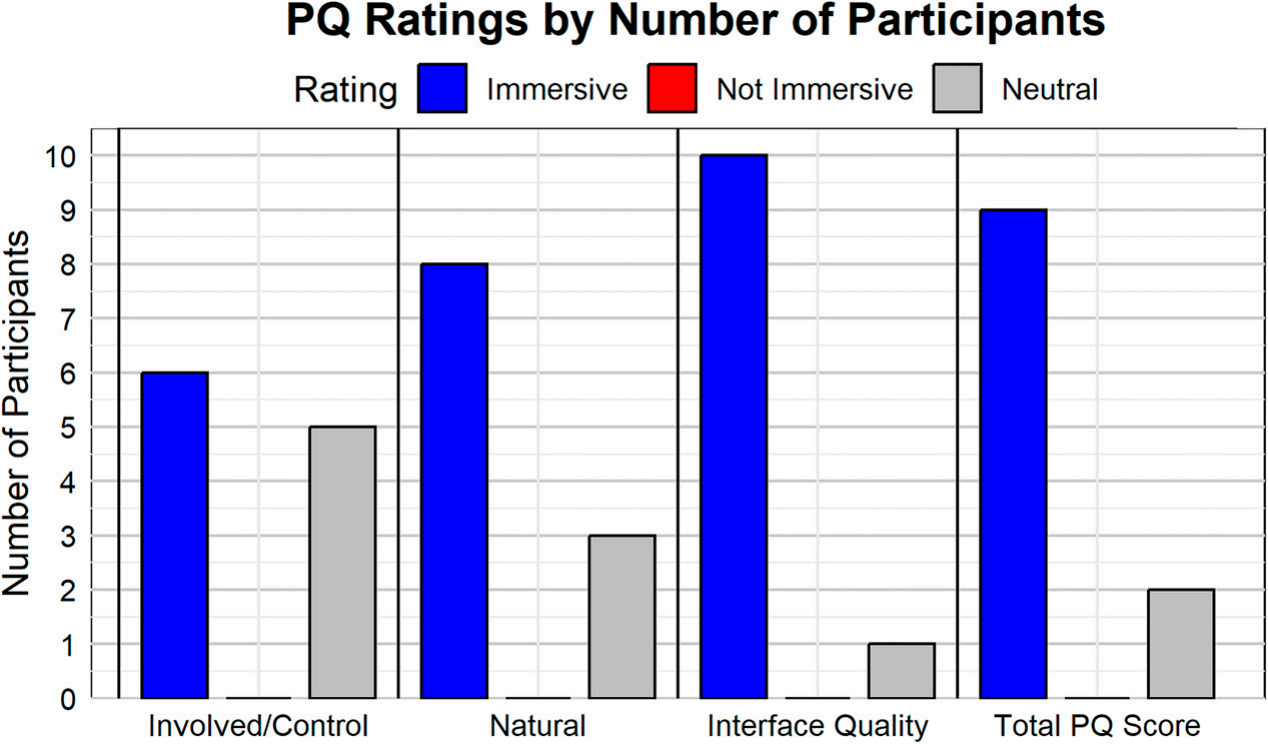
Describing the number of participants who gave an “immersive,” “not immersive,” or “neutral” rating of the intervention for each of the subscales in the Presence Questionnaire (PQ).

Overall, the UES summary scores suggest five participants found the VR GMI program engaging (45.45%), five participants were neutral about their level of engagement (45.45%), and one participant did not find the VR GMI program engaging (0.09%). A similar trend was observed across the UES subscales, with the exception of focused attention (where over half of the participants endorsed neutral engagement; 72.73%) and perceived usability/reward (in which over half of participants rated the program favorably; 81.81% and 72.73%, respectively). Meanwhile, nine participants (81.81%) rated the immersion of the VR GMI program favorably, as indicated by their PQ summary and subscale scores. See Figures 4 and 5 for bar graphs illustrating the number of participants associated with each subscale on the UES and PQ scales.

## Discussion

Even though most people with LLAs experience PLP most severely immediately following LLA, effective, acute treatment is currently inaccessible.^1,18^ The present study represents the first multi-method design to evaluate the feasibility of a VR program that facilitates the self-administration of GMI to increase treatment access and improve health outcomes.

**FIG. 6.**
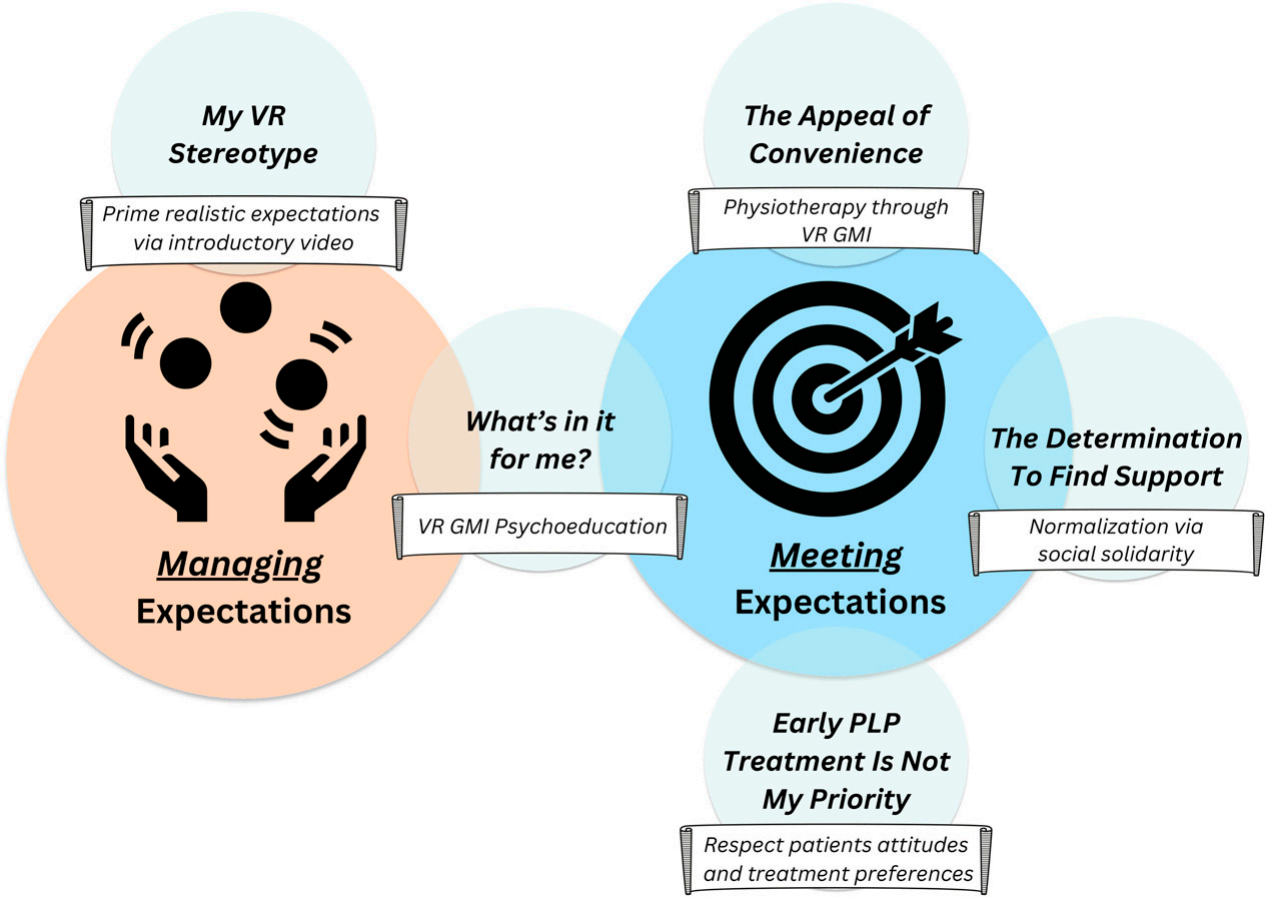
Providing a visual overview of each of the five themes and how they relate to the main topics: managing expectations and meeting expectations. Development priorities are listed under each of the corresponding themes. VR, virtual reality; GMI, graded motor imagery.

Participants in the current study rated the VR GMI program favorably for its capacity to induce feelings of immersion, in addition to its perceived usability, and judged it to be an overall rewarding experience. However, most participants expressed neutral feedback regarding the VR GMI program’s capacity to focus their attention. These results suggest future development of the VR GMI program needs to more adequately engage the attention of the user. In the current study, qualitative data were also collected and analyzed to elucidate barriers and facilitators that may deter or promote use of the VR GMI intervention to treat PLP. The resulting themes suggest motivation to engage with the VR GMI program is strongly influenced by individual expectations of what VR and rehabilitative treatment should entail. Specifically, VR GMI program engagement could be improved in two ways: by managing expectations and by meeting expectations. See Figure 6 for a visual map of these topics and their affiliated themes.

### Managing VR GMI expectations: The importance of psychoeducation

The themes *My VR stereotype* and *What’s in it for me?* underscore the need to manage expectations before introducing patients to the VR GMI program. Participants expressed reservations about using VR independently, often linking their doubts to age-related capability concerns. This aligns with findings that older patients are less inclined than younger ones to participate in VR interventions due to a confidence gap termed the digital divide.^29^ Cost concerns also arose (in line with previous literature), though decreasing VR costs and potential government support could help mitigate this barrier.^14,30^ Despite these reservations, most participants reported positive engagement and immersion ratings with the VR GMI program, with engagement largely influenced by the technology’s alignment with their expectations. For instance, P11 reported high engagement because his expectations—that VR is like the simulation demonstrated in the movie *Avatar*—were met, whereas P7, perceiving the graphics as “amateurish” and “blocky,” rated his engagement low due to unmet expectations. Embedding psychoeducation at the outset of VR GMI can help set more realistic expectations and demonstrate the program’s immediate and preventative benefits, encouraging patient buy-in while simultaneously emphasizing that the program has been developed for people of all ages.^3,12^ For people with LLAs, who often feel helpless in managing PLP, structured psychoeducation could foster greater confidence and autonomy in VR GMI use.^31–34^

### Meeting VR GMI expectations: The importance of patient-centered care

While psychoeducation can prime realistic expectations, the qualitative themes also allude to the importance of patient-centered care. Patient-centered care involves prioritizing individuality and empowerment in health care by respecting the active role patients play in their treatment.^35^ Through this perspective, development should not only focus on *managing* patient expectations but also focus on *meeting* patient expectations. For example, participants often expressed interest in integrating traditional physiotherapy into the VR GMI program. More holistic PLP treatment through the VR GMI program could entail simultaneous treatment of physical and phantom pain, such as prompting the user to increase one’s number of exercise repetitions in the limb simulation stage. Additionally, participants indicated neutral ratings of the program’s aesthetic appeal. Aesthetic expectations could be more adequately met by incorporating specific participant requests, such as relaxing natural scenery during explicit motor imagery and including settings beyond that of a hospital room in the limb simulation stage. Lastly, the VR GMI program could also facilitate social connection among people with LLAs. Previous research suggests most people with LLAs are dissatisfied with the level of support provided by health care professionals in their transition from inpatient to outpatient care and desire a facilitated connection with fellow amputees.^15,17,32,36,37^ The VR GMI program could incorporate social support by embedding videos of people with LLAs talking to the user about how they coped and succeeded following amputation, as recommended by participants. Holistic health care considerations may bolster VR GMI program adherence by attending to other priorities in addition to PLP.

### Strengths and limitations

The current study employed a rigorous multi-method design suited for intervention development. The primary researcher (M.C.) was immersed in the study from data collection to interpretation, maximizing her data familiarization in line with recommendations from the creators of reflexive thematic analysis.^22,23^ Qualitative sensibility was achieved through close collaboration with qualitative researchers throughout the analysis. Psychometrically validated self-report measures were employed to enhance the rigor of the qualitative findings through methodological triangulation. Importantly, the current research follows a framework to validate the VR GMI program through a user-centered approach.^38^ These strengths enhance the validity of the findings.

The present research also presents some limitations. Participants only completed one brief session with the VR GMI program before speculating as to what may motivate or deter their use of it; administration in the acute care setting may yield different barriers. Additionally, a purposive sampling procedure was employed, yielding a sample that does not represent the population of individuals receiving LLAs in Manitoba (e.g., Indigenous peoples receiving LLAs due to diabetic complications).^39^ For these reasons, the current findings should not be generalized.

## Implications and Conclusion

The implications of the current research are threefold. First, this is one of the first studies in the VR PLP literature to prioritize clinical implementation through a validation framework, which is recommended to increase the clinical implementation of VR interventions.^40^ Accordingly, the VR GMI program will also be assessed through large feasibility studies and randomized controlled trials conducted in the hospital and patient homes in the acute period of recovery following amputation. Second, this study contributes to a novel field of research investigating the potential of acute PLP treatment using VR. Recently, a group of researchers from India demonstrated a significant treatment effect on PLP intensity at 6 months following administration of mirror therapy for 2 weeks immediately following LLA.^41^ The VR GMI program aims to build on this research not just by establishing the efficacy of acute PLP treatment but also its feasibility. Lastly, the VR GMI program presents people with LLAs the opportunity to bypass barriers to PLP treatment. With this intervention, people with LLAs would be able to treat their PLP on their own terms, immediately following their LLA if they so choose. The findings may also have broader implications for consideration of the implementation of VR treatments in perioperative settings, particularly the acute postoperative period.

## Abbreviations Used

GMI: Graded motor imagery
LLA: Lower limb amputation
NRC: National Research Council
PLP: Phantom limb pain
PQ: Presence Questionnaire
UES: User Engagement Scale
VR: Virtual reality
