# Supplementary material for: Anticipated Barriers and Facilitators to Engaging in a Novel Virtual Reality Program for Lower Phantom Limb Pain
Source: J Med Ext Real. 2025 Mar 31;2(1):61–75. doi: 10.1089/jmedxr.2024.0047 (PMC13387243; doi:10.1089/jmedxr.2024.0047)
Supplement: Supplementary file 1 [file jmedxr.2024.0047_supplementary_appendix.docx]

**Appendix A**

Written Description of the Virtual Reality (VR) Graded Motor Imagery (GMI) Program

***Left/Right Discrimination***

The first treatment task was left/right discrimination. The participant was instructed to sort a series of images of feet in different orientations. Participants sorted ten images of each of the following image types: basic (plain background), vanilla (more than just the foot in the photo [e.g., calf], not necessarily a plain background), and context (flipped to varying degrees, foot is seen doing a variety of tasks seen in everyday life). The images were displayed on pieces of toast that burned when they sorted an image incorrectly. At the end of each image set, the speed and accuracy with which they sorted the images was displayed.

***Explicit Motor Imagery***

The second VR stage was explicit motor imagery, where the participant imagined (rather than actually performed) a series of movement with their phantom leg. To begin this stage, the participant underwent a relaxation induction and body scan exercise that facilitated focus on the phantom limb, during which time the VR interface was turned off. Following the body scan, the participant underwent the same process for two different exercises. The participant first watched a virtual clinician perform the exercise. Then, the screen went black and the participant imagined performing the exercise themselves. Audio described the sensations the participant may notice as they perform each exercise, with the intention of facilitating focus on the phantom limb.

***Limb Simulation***

In the final stage of the VR, the participant executed movements with their phantom leg while receiving virtual visual feedback of the action through the VR program. First, the participants went through six exercises. The virtual clinician (who appeared in a virtual hospital room) showed the participant what each exercise looked like, and a countdown played when it was time for the participant to try. Both legs of the self-avatar moved on screen; the participant mimicked these movements with their intact leg and imagined symmetrical movements with their phantom limb. This level of the mirror therapy stage was called “standard mode” because the participants performed the tasks at a default speed, number of repetitions, and order. After trying the standard exercises, the participants were permitted to engage with the exercises in any order, at varying speeds, and with varying repetitions for each set. The participants were able to engage in these exercises while controlling the order, number of repetitions, and speed they played at.

If time permitted, participants trialled limb simulation with leg trackers. In this mode, straps were put on the participants’ legs that allowed the *Oculus Quest 2* controllers to attach to their lower limbs (just above their knees, or the lowest part available on their limb). Participants were then able to control their virtual legs without needing to wait for the self-avatar to move on its own. This was intended to increase one’s sense of agency over the virtual legs.
